# Supplementary figures and images for: Fluorescent protein tags for human tropomyosin isoform comparison
Source: Biol Open. 2025 Jul 31;14(8):bio061992. doi: 10.1242/bio.061992 (PMC12352278; doi:10.1242/bio.061992)

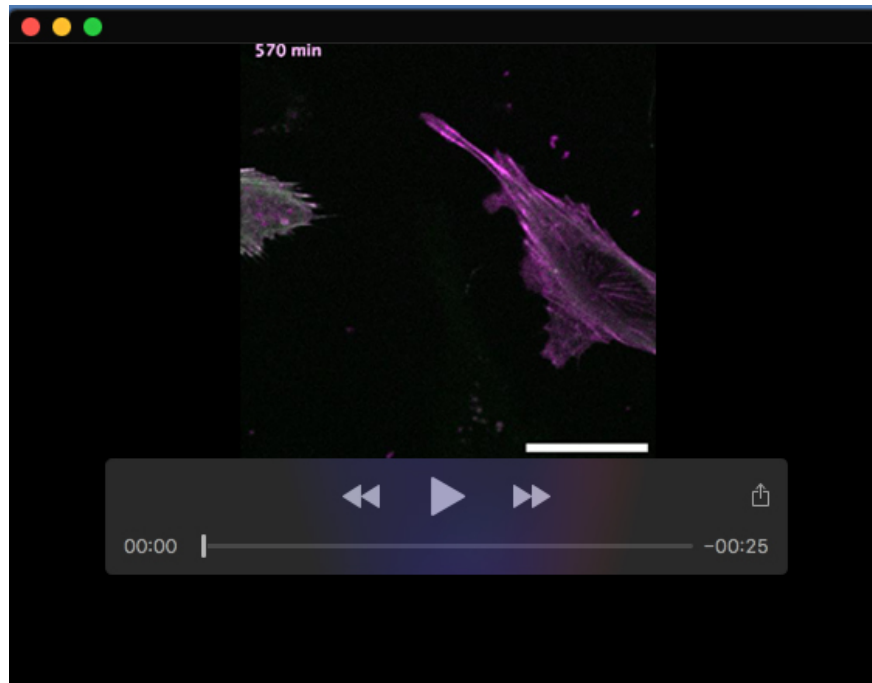

**Movie 1.**

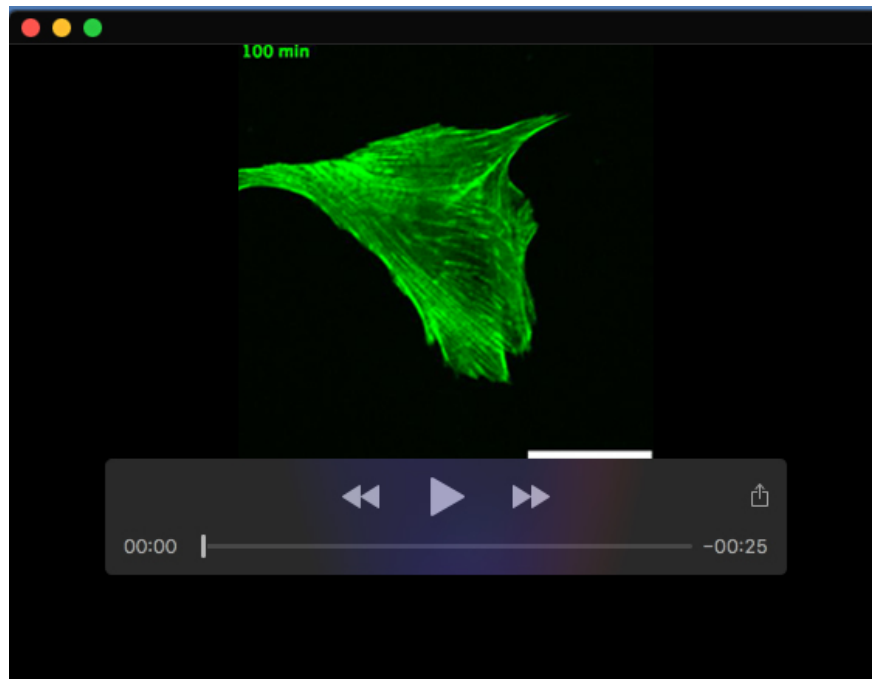

**Movie 2.**

Supplement: Supplementary information [file biolopen-14-061992-s1.pdf]
